# Maximizing the general success of cecal intubation during propofol sedation in a multi-endoscopist academic centre

**DOI:** 10.1186/1471-230X-10-123

**Published:** 2010-10-20

**Authors:** Fabrizio Cardin, Nadia Minicuci, Alessandra Andreotti, Elena Pinetti, Federico Campigotto, Barbara M Donà, Bruno Martella, Oreste Terranova

**Affiliations:** 1 Geriatrics Department, Geriatric Surgery Unit, General Hospital, Padova, Italy; 2 National Research Council, Institute of Neuroscience, Padova Section, Italy; 3 Department of Pharmacology and Anesthesiology, Anesthesia and Intensive Care Unit, Padova, Italy; 4 Veneto Oncologic Institute, IRCCS, Padova, Italy

## Abstract

**Background:**

Achieving the target of 95% colonoscopy completion rate at centres conducting colorectal screening programs is an important issue. Large centres and teaching hospitals employing endoscopists with different levels of training and expertise risk achieving worse results. Deep sedation with propofol in routine colonoscopy could maximize the results of cecal intubation.

**Methods:**

The present study on the experience of a single centre focused on estimating the overall completion rate of colonoscopies performed under routine propofol sedation at a large teaching hospital with many operators involved, and on assessing the factors that influence the success rate of the procedure and how to improve this performance, analyzing the aspects relating to using of deep sedation. Twenty-one endoscopists, classified by their level of specialization in colonoscopic practice, performed 1381 colonoscopies under deep sedation. All actions needed for the anaesthesiologist to restore adequate oxygenation or hemodynamics, even for transient changes, were recorded.

**Results:**

The "crude" overall completion rate was 93.3%. This finding shows that with routine deep sedation, the colonoscopy completion rate nears, but still does not reach, the target performance for colonoscopic screening programs, at centers where colonoscopists of difference experience are employed in such programs.

Factors interfering with cecal intubation were: inadequate colon cleansing, endoscopists' expertise in colonoscopic practice, patients' body weight under 60 kg or age over 71 years, and the need for active intervention by the anaesthesiologist. The most favourable situation - a patient less than 71 years old with a body weight over 60 kg, an adequate bowel preparation, a "highly experienced specialist" performing the test, and no need for active anaesthesiological intervention during the procedure - coincided with a 98.8% probability of the colonoscopy being completed.

**Conclusions:**

With routine deep sedation, the colonoscopy completion rate nears the target performance for colonoscopic screening programs, at centers where colonoscopists of difference experience are employed in such programs. Organizing the daily workload to prevent negative factors affecting the success rate from occurring in combination may enable up to 85% of incomplete procedures to be converted into successful colonoscopies.

## Background

In colorectal cancer screening, successful colonoscopy is related to the polyps detection rate and the percentage of complete colon examinations achieved with cecal intubation. Both factors depend on technical issues and the endoscopist's performance [1]. The quality standard for the polyps detection rate (set at 25% in men and 15% in women) is related to the withdrawal time and differences seem to be due more to endoscopists' individual sensitivity than to their technical expertise [2].

Completed colonoscopy rates of 97-99% have been reported and, although a rate of 90% is acceptable in routine clinical activity, it is best to aim for at least 95% of completed colonoscopies in screening programs [3]. Various technical factors play a part, such as the calibre [4] or stiffness [5] of the instrument used, and the time of day when the test is scheduled [6], but the endoscopists' experience and the number of endoscopies they have already performed have an important influence on the success of the test [7].

Some endoscopy units achieve good results because the activity is handled by a single, dedicated endoscopist [8,9], while large teaching centres reach lower quality indicators for their colonoscopy activities [10]. In large hospitals, the overall colonoscopy completion rates are influenced by the procedure being implemented by trainee doctors and by endoscopists from different specialities (gastroenterological, surgical, or internal medicine). In such cases, strategies have been proposed to improve performance based on auditing programs, adjusting the various endoscopists' workload in the light of their results [11], or having trainees use auxiliary devices to facilitate cecal intubation [12].

In hospitals with a large number of practising colonoscopists, a rapid strategy to optimize global performance and achieve an acceptable number of complete colonoscopies with minimal patient discomfort could be to routinely use deep sedation with propofol. This is considered a procedure with an acceptable safety profile [13], but the extensive use of such a method to improve technical results is a debatable issue [14]. The aim of this study was to present the results of routine propofol sedation for colonoscopy at a large teaching hospital where a large number of doctors with differing levels of expertise are involved in colonoscopy activities. We also assessed the factors influencing the success rate of the procedure and ways to improve performance, analyzing the complementary aspects relating to the use of deep sedation.

## Methods

We investigated the routine activities at an endoscopy centre at Padova University teaching hospital, consecutively enrolling all patients who had a colonoscopy under deep sedation in the study over a period of one year (February 2007 to February 2008).

Subject to patients' informed written consent, their clinical history was recorded to identify any anaesthesiological or procedural risks, collecting data on: a) non-gastrointestinal conditions; b) allergies or side effects of previously-used anaesthetics; c) ASA status [15]. Information was then recorded on the completeness of the colonoscopy, the propofol dosage used and any adverse events on a case report form (CRF) formulated specifically for the purposes of the study. The health professionals responsible for compiling the CRF were unaware that they were participating in the study. Patients were not enrolled in the study if they refused to sign the consent form or if they were willing to undergo colonoscopy without sedation, or if the endoscopist preferred for clinical or technical reasons to perform the colonoscopy without sedation (e.g. as a follow-up procedure in a patient who had already tolerated the test well, or in a patient with an operated colon).

### Institutional review board approval

According to the guidelines of Italian Agency of Drugs (AIFA) the observational studies which use retrospective data or materials do not require formal approval by the local Ethics Committee.

### Endoscopist classification

The endoscopists involved in the colonoscopy procedures were classified according to their level of experience, grouped into three categories, defined as: 1) "less experienced non-specialists" if they had been performing endoscopies for less than 10 years; 2) "more experienced non-specialists" if they had been performing endoscopies for more than 10 years but were not exclusively dedicated to endoscopic activities (performing up to two endoscopic sessions a week); and 3) "highly experienced specialists" if they had been performing endoscopies for more than 10 years and handled at least four endoscopic sessions a week (the scheduled number of colonoscopies per session is four). There were three gastroenterologists involved; all the other operators were surgeons.

### Propofol sedation

Before colonoscopy, all patients received an initial induction dose of propofol (0.5-1 mg/Kg) to induce a lethargic response to oral stimuli and no corneal reflex. During the procedure, propofol was titrated by the anaesthetist with intermittent boluses if patients showed signs of more than mild discomfort and occasional grimacing, or became agitated or were clearly in pain at any stage of the procedure.

Patient monitoring was started before sedation and continued until patients recovered to check for any episodes of hypotension, hypoxemia or cardiac arrhythmia, which were recorded in the CRF and treated pharmacologically, where necessary.

The patients' preparatory colon cleansing consisted in ingesting four litres of Macrogol solution at home on the day before the colonoscopy. Their bowel preparation was classified as: 1) "adequate", 2) "with residual matter" or 3) "inadequate" when faeces prevented the continuation of the examination.

### Definition of complete colonoscopy

The main efficacy endpoint was the completeness of the colonoscopy, defined as the identification of a normal cecal anatomy or ileocolic anastomosis. When colonoscopy was interrupted due to organic stenoses, it was considered as complete, as stated by Rex et al. (3). Any anaesthesiological, pharmacological or manual measures taken by the anaesthetist were recorded and classified as "active anaesthesiological intervention" (AAI).

Hemodynamic monitoring data were used to determine the duration of the colonoscopy.

### Sample size determination

In the literature[3], the colonoscopy completion rate is reportedly around 97%. The PASS 2008 software was used to determine the sample size needed for our study, which was identified as a sample size of 1216 in order to produce a two-sided 95% confidence interval with a width of 0.020 (CI 96%-98%) when the sample proportion is 0.970. Assuming a 20% drop-out rate, 1500 records were analyzed vis-à-vis the inclusion criteria.

### Statistical analysis

Summary statistics (mean values or percentages) were compared by completeness-of-colonoscopy groups using the *t*-test (after checking the homoschedasticity) and the chi-square (χ^2^) test, respectively. If they were not applicable, analogous non-parametric tests were used (Wilcoxon's rank-sum test or Fisher's exact test).

Predictors of an incomplete colonoscopy were investigated by multivariate stepwise (p-entry = 0.15) logistic regression analysis, including sex, age, body weight, ASA category, total dosage of propofol administered, duration of the procedure, endoscopists' experience levels, bowel preparation, and any AAI as predictors.

The SAS statistical software, rel. 9.1.3, was used for the analysis. A p-value < 0.05 was used to establish statistical significance.

## Results

During the study period, a total of 2,027 colonoscopies were performed, 1,381 of them under deep sedation and the latter formed the object of this study (Table 1). The incomplete procedures amounted to 92 and 67 of these were incomplete for technical reasons, 25 due to inadequate bowel preparation. Older age was statistically associated with incomplete colonoscopy (64.2 vs 60.5 years)

**Table 1:** Distribution of the main characteristics by completeness of colonoscopy

	Complete colonoscopy		P value
		
	Yes (n = 1289)	No (n = 92)	
Sex (%)			Ns
Female	53.4%	54.3%	

Age			0.007
Mean ± SD	60.5 ± 13.6	64.2 ± 15.3	
Range	14-95	21-91	

Body Weight (kg)			Ns
Mean ± SD	71.7 ± 14.1	69.8 ± 15.5	
Range	37-135	42-120	

ASA (%)			0.002
1	38.3%	36.9%	
2	53.9%	44.6%	
3+4	7.8%	18.5%	

Propofol (mg)			0.02
Mean ± SD	191.2 ± 78.3	172.1 ± 91.6	
Range	30-650	20-430	

Duration of procedure (minutes)			Ns
Mean ± SD	24.4 ± 11.1	23.8 ± 13.4	
Range	5-85	4-70	

O2 l/m (%)			Ns
≤ 4 l/m	95.4% (n = 992)	96% (n = 72)	
> 4 l/m	4.6% (n = 48)	4% (n = 3)	
Missing (n)	249	17	

SPO2			Ns
Mean ± SD	98.54 ± 1.73	98.46 ± 1.77	
Range	80-100	89-100	
Missing (n)	83	6	

Presence of AAI (%)			0.02
Yes	5.2%	10.9%	

Endoscopists (n = 21) (%)			< 0.0001
Less experienced non-specialists (n = 7)	9.7%	25.0%	
More experienced non-specialists (n = 8)	66.1%	66.3%	
Highly experienced specialists (n = 6)	24.2%	8.7%	

Bowel preparation (%)			< 0.0001
Adequate	72.7%	46.7%	
With residual matter	24.1%	26.1%	
Inadequate	3.2%	27.2%	

Polypectomy (%)			0.03
Yes	17.3%	8.7%	

There were 77 AAI during the procedures (5.2% among the complete colonoscopies and 10.9% among the incomplete colonoscopies; p-value = 0.02) to deal with 28 respiratory(solved using mask ventilation, without any need for intubation) and 39 hemodynamic problems, 8 episodes of regurgitation, and 2 of allergic reactions (the patients were simultaneously treated with antibiotic prophylaxis). We observed two complications of the endoscopic procedure, i.e. one perforation and one post-polypectomy hemorrhage. Polypectomy was performed during 231 procedures.

A statistically significant association emerged between ASA scores and completeness of colonoscopy (p-value = 0.002): 53.9% of patients with completed colonoscopies and 44.6% of those with incomplete colonoscopies had an ASA score of 2; the corresponding percentages among the patients with ASA scores of 3 or 4 were 7.8% and 18.5%.

The mean total doses of propofol administered differed statistically between the two groups with a p-value of 0.02 (191.2 mg vs 172.1 mg, respectively).

The endoscopists' levels of experience were unevenly distributed between the two groups, with a greater prevalence of highly experienced specialists associated with the completed colonoscopy group (24% vs 8.7%, p-value < 0.0001).

Sex, body weight, duration of the procedure, O2 l/m and SPO2 variables were not associated with the completeness of colonoscopy.

About 73% of patients with completed colonoscopies had adequately prepared bowels, 24% of patients had residual matter and 3% of patients had inadequately cleansed bowels; the corresponding percentages among patients with incomplete colonoscopies were: 47%, 26% and 27%, revealing a statistical association (p-value < 0.0001).

"Less experienced non-specialist" endoscopists were also more frequently associated with cases of inadequate bowel preparation (Table 2).

**Table 2:** Experience level of the endoscopists versus the bowel preparation (N)

	Bowel preparation			
		
Endoscopist	Adequate	Presence of residual matter	Inadequate	Total
Less experienced non-specialist	95	36	17	148
More experienced non-specialist	632	240	41	913
Highly experienced specialist	253	59	8	320

Total	980	335	66	1381

χ^2 ^p-value < 0.0001

Logistic regression analysis (Table 3) showed that patients with inadequately prepared bowels had an 11-fold increase in the risk of incomplete colonoscopy by comparison with patients with an adequate bowel preparation, while the risk associated with the presence of residual matter was not significant. Having a colonoscopy done by a less experienced endoscopist coincided with a 5-fold increase in the risk of an incomplete procedure by comparison with colonoscopies handled by a highly experienced specialist; this risk dropped to a 2-fold increase if the test was performed by a more experienced non-specialist.

**Table 3:** Odds ratios and 95% CI for incomplete colonoscopies

	Odds Ratio	95% CI	P value
Age ≥ 71 years old	2.09	1.31-3.32	0.002
Body weight < 60 kg	1.89	1.14-3.11	0.013
Highly experienced specialist	1		
More experienced non-specialist	2.47	1.15-5.34	0.021
Less experienced non-specialist	5.19	2.17-12.41	0.0002
Adequate bowel preparation	1		
Presence of residual matter	1.59	0.94-2.68	0.08
Inadequate bowel preparation	10.81	5.86-19.94	< 0.0001
Presence of AAI	2.79	1.21-6.38	0.015

The need for AAI and age over 71 years coincided with a 2.7-fold and a 2.1-fold increase in the risk of a colonoscopy not being completed; weighing less than 60 kg also emerged as a risk factor (OR = 1.89).

The logistic model had an area under the ROC curve of 77.8% and the value of the Hosmer-Lemeshow goodness of fit statistic was 2.05, with a corresponding p-value of 0.91, indicating that the model seems to fit quite well. None of the other variables contributed to the predictive power of the model.

To gain a better understanding of the contribution of the various factors involved in the completion of a colonoscopy, the logistic regression model was used to calculate the probabilities of complete colonoscopy according to different combinations of values of these predictors. The most favourable situation - a patient less than 71 years old with a body weight over 60 kg, an adequate bowel preparation, a "highly experienced specialist" performing the test, and no need for AAI during the procedure - coincided with a 98.8% probability of the colonoscopy being completed. The probabilities related to all other possible combinations (71 in all) of the values of these predictors were computed and the % variations between each combination and the most favourable situation were calculated. For example, the second most favourable situation (a patient less than 71 years old with a body weight over 60 kg, an adequate bowel preparation, a "more experienced non-specialist" performing the test, and no need for AAI during the procedure) carried a probability of successful colonoscopy of 96.7% with a 1.7% variation with respect to the most favourable situation. This means that the involvement of a "more experienced non-specialist" (symbol E, Table 4) reduces the probability of success by nearly 2%. If we consider the case of a combination of a less experienced non-specialist and the need for AAI (EEA), the difference increases to 14.2%. On the other hand, if the patient's bowel preparation is also inadequate, these same two factors (EEA) reduce the probability of success by 65%.

**Table 4:** Description of the symbols used in the Figure 1

Symbol	Description
E	Presence of a more-experienced non specialist (vs highly-experienced specialist)
A	Presence of AAI (vs none)
EE	Presence of a less-experienced non specialist (vs highly-experienced specialist)
EA	Presence of a more-experienced non specialist and AAI (vs highly-experienced specialist and no AAI)
EEA	Presence of a less-experienced non specialist and AAI (vs highly-experienced specialist and no AAI)

**Figure 1:**
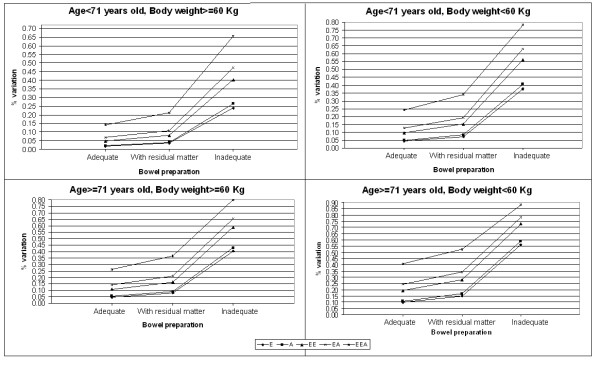
**Percentage variations in colonoscopy success rates by patients' characteristics**. This figure shows the percentage variations in colonoscopy success rate by level of experience of the endoscopists, age of the patient, body weight of the patients and bowel preparation.

These findings are graphed in Figure 1 by patient's age and body weight categories (unmodifiable predictors) and bowel preparation.

The greatest variation (more than 85%) is recorded for a patient over 71 years old with a body weight below 60 kg and inadequate bowel preparation, needing AAI during a procedure conducted by a less experienced non-specialist.

## Discussion

It has already been emphasized that the colonoscopy completion rate is important for more than just academic reasons [8] and one important reason is that a completed colonoscopy reduces the likelihood of an advanced colorectal-cancer being detected later on [16].

In this report on a single centre's experience of colonoscopies systematically associated with deep sedation with propofol, the global success rate achieved by 21 endoscopists working at the same endoscopic centre with different levels of expertise and training was 93%.

The factors negatively influencing the success of the test were: a patient's small body size, age over 71 years, inadequate colonic cleansing and the need for AAI during deep sedation. For the purposes of this study, we did not correct the performance rate by excluding colonoscopies in which cecal intubation had been prevented by the presence of faecal material, because our aim was to establish the crude success rate for colonoscopy under deep sedation. It is worth adding here that more tests were interrupted due to poor cleansing issues when less expert endoscopists were involved.

Two complications of endoscopy (one perforation and one post-polypectomy haemorrhage) were recorded during the study period, a prevalence similar to that of other reports [17]. We used a broad definition of "adverse events" during colonoscopy under deep sedation, recording all action taken by the anaesthetist to restore adequate oxygenation or hemodynamics, even for short-lived monitoring problems, because such situations might influence the procedure or have to do with longer probe insertion times necessitating further propofol infusions. No hemodynamic resuscitation was needed and there were no major clinical sequelae after sedation.

One of the limits of this study is that we were not in a position to compare how different colonoscopic methods might improve the colonoscopy completion rate.

Although ours was an observational study on the controversial issue of the routine use of deep sedation for colonoscopies, it had the advantage of referring to a recognized target (i.e. 95% cecal intubations in colonoscopies for screening purposes). Other published series adopted a different approach, achieving a different gap between their results and the 95% target [18-20].

The performance measurement showed that the endoscopist's skill influences the success of colonoscopies even when deep sedation is used. In a clinical setting, this poses problems that have already been studied and overcome using quality improvement programs and auditing cycles [11]. All our endoscopists had gained experience during weekly sessions for more than a year, implementing 200 procedures alone (a number recognized as being sufficient to achieve a satisfactory performance in screening programs)[21]. When it comes to screening programs, the endoscopists' different levels of experience carry a different weight from the situation in routine clinical practice because the most important problem is often the shortage of operators. Solutions have to be found to deal with the problems of the burden of colonoscopies to perform and increasingly long waiting lists, which is why colonoscopies may be performed by gastroenterologists, clinical assistants, trainee gastroenterologists or nurses in some countries [22].

The aim of our study, however, was to ascertain whether using propofol sedation enables the standard to be reached in screening programs, when it is necessary to employ many endoscopists who are likely to have different levels of expertise.

Success rate has been seen as an expression of "technical machismo" [8] and some authors suggest that it should not be influenced by patient-related factors and that, in any case, a trained endoscopist should be able to complete 95% of colonoscopies successfully [23]. We considered it important to demonstrate that patients' weight and age, as already mentioned in other studies [24,25] without deep sedation, are factors that also predict incomplete colonoscopies among series of colonoscopies performed under deep sedation at a busy teaching hospital service, where endoscopists of different abilities are at work.

Different strategies can be used to improve the quality of colonoscopic screening programs. We chose to consider the role of routine deep sedation. Propofol was preferred as a sedative because previous observational studies using other drugs for sedation during colonoscopy in almost 94% of the sample had still reported unacceptably low success rates [26]. In addition, it has recently been demonstrated that deep sedation enhances the polyps detection rate [27].

A weakness of our study is that we did not perform a thorough cost-benefit analysis, particularly as concerns the need for extra personnel to manage sedation. This issue depends largely on differences in the compensation awarded by national health systems and on their related organizational aspects, but relevant data could probably be obtained quite easily by adapting our findings to different situations in different countries. We also collected no details on patients' satisfaction with the procedure, because this information is only collected at our centre in the context of clinical trials and we wished to avoid any Hawthorne effect on the endoscopists' routine practice.

Our study has shown a possible strategy for further improving the rate of successful cecal intubations, i.e. by modifying certain organizational aspects when deep sedation is used.

If a "difficult patient" (aged over 71 and weighing less than 60 kg) has to undergo colonoscopy at the hands of a "less experienced non-specialist" (instead of a "highly experienced specialist"), then inadequate colon preparation reduces the chances of the colonoscopy being completed by 70%. So, special attention should be paid to colon-cleansing practices for colonoscopies that are to be handled by a less experienced endoscopist. Moreover, if any unwanted effects of propofol infusion demanding AAI can be avoided by a careful management of sedation, then 60% of incomplete procedures could be successful. In other words, if a difficult colonoscopy is programmed (due to the patient's characteristics), then containing the need for AAI and improving bowel cleansing enables the successful completion of 90% of incomplete colonoscopies whatever the expertise of the endoscopist involved.

## Conclusions

In conclusion, where there are workforce shortages and at large endoscopic practices where colonoscopies are handled by doctors with different levels of training and experience, the extensive use of propofol enables the standard success rate for screening colonoscopies to be approached. An excellent result can be achieved, in terms of the completeness of the procedures, especially if deep sedation is combined with a careful preliminary assessment of the workload so that patients listed for endoscopic procedures who might prove particularly difficult to handle (a situation that is readily identifiable from simple, known features) are not assigned to endoscopists without a high level of specialist experience.

## Abbreviations

ASA: American Society of Anesthesiologists; CRF: Case Report Form; AAI: Active Anesthesiological Intervention; CI: Confidence Interval; OR: Odds Ratio, ROC: Receiver Operating Characteristic

## Competing interests

The authors declare that they have no competing interests.

## Authors' contributions

FC, OT, EP, and BMD performed the conception and the design of the study.

FC, NM, AA, and FC performed the statistical analysis and the interpretation of the findings.

FC, NM, and AA performed the drafting of the article. BM performed a critical revision of the article for important intellectual content.

All authors read and approved the final manuscript.

## Pre-publication history

The pre-publication history for this paper can be accessed here:

http://www.biomedcentral.com/1471-230X/10/123/prepub
